# Faster algorithms for RNA-folding using the Four-Russians method

**DOI:** 10.1186/1748-7188-9-5

**Published:** 2014-03-06

**Authors:** Balaji Venkatachalam, Dan Gusfield, Yelena Frid

**Affiliations:** 1 Department of Computer Science, University of California, Davis, 1 Shields Ave, Davis, CA, USA

**Keywords:** RNA-folding, Four-Russians, CUDA, Algorithms, Parallel algorithm, GPU

## Abstract

**Background:**

The secondary structure that maximizes the number of non-crossing matchings between complimentary bases of an RNA sequence of length *n* can be computed in *O*(*n*^3^) time using Nussinov’s dynamic programming algorithm. The Four-Russians method is a technique that reduces the running time for certain dynamic programming algorithms by a multiplicative factor after a preprocessing step where solutions to all smaller subproblems of a fixed size are exhaustively enumerated and solved. Frid and Gusfield designed an $O(\frac{n^{3}}{\log n})$ algorithm for RNA folding using the Four-Russians technique. In their algorithm the preprocessing is interleaved with the algorithm computation.

**Theoretical results:**

We simplify the algorithm and the analysis by doing the preprocessing once prior to the algorithm computation. We call this the *two-vector* method. We also show variants where instead of exhaustive preprocessing, we only solve the subproblems encountered in the main algorithm once and memoize the results. We give a simple proof of correctness and explore the practical advantages over the earlier method.

The Nussinov algorithm admits an *O*(*n*^2^) time parallel algorithm. We show a parallel algorithm using the two-vector idea that improves the time bound to $O(\frac{n^{2}}{\log n})$.

**Practical results:**

We have implemented the parallel algorithm on graphics processing units using the CUDA platform. We discuss the organization of the data structures to exploit coalesced memory access for fast running times. The ideas to organize the data structures also help in improving the running time of the serial algorithms. For sequences of length up to 6000 bases the parallel algorithm takes only about 2.5 seconds and the two-vector serial method takes about 57 seconds on a desktop and 15 seconds on a server. Among the serial algorithms, the two-vector and memoized versions are faster than the Frid-Gusfield algorithm by a factor of 3, and are faster than Nussinov by up to a factor of 20. The source-code for the algorithms is available at http://github.com/ijalabv/FourRussiansRNAFolding.

## Background

Computational approaches to find the secondary structure of RNA molecules are used extensively in bioinformatics applications.

The classic dynamic programming (DP) algorithm proposed in the 1970s has been central to most structure prediction algorithms. While the objective of the original algorithm was to maximize the number of non-crossing pairings between complementary bases, the dynamic programming approach has been used for other models and approaches, including minimizing the free energy of a structure. The DP algorithm runs in cubic time and there have been many attempts at improving its running time [1,2]. Here, we use the Four-Russians method for speeding up the computation.

The Four-Russians method, named after Aralazarov et al. [3], is a method to speed up certain dynamic programming algorithms. In a typical Four-Russians algorithm there is a preprocessing step that exhaustively enumerates and solves a set of subproblems and the results are tabled. In the main DP algorithm, instead of filling out or inspecting individual cells, the algorithm takes longer strides in the table. The computation for multiple cells is solved in constant time by utilizing the preprocessed solutions to the subproblems. The longer strides to fill the table reduce the runtime by a multiplicative factor. The size of the subproblems is chosen in a way that does not make the preprocessing too expensive.

Frid and Gusfield [4] showed the application of the Four-Russians approach for RNA folding. In their algorithm, the preprocessing is interleaved with the algorithm computation. They fill out a part of the DP table and use these entries to complete a part of the preprocessing. The preprocessed entries are used later in the computation.

We show a simpler algorithm, where, all the preprocessing is completed before the start of the main algorithm. This simplifies the correctness proof and the runtime analysis. This approach helps in obtaining an *O*(*n*^3^/ log*n*) time parallel algorithm, which is a log*n* factor improvement over previously-known parallel algorithms. Zakov and Frid (personal communication) had also observed that the algorithm in [4] could be modified to do the preprocessing once at the start of the algorithm. It is essentially the idea described here.

In this paper we explore the implications of the one-pass preprocessing idea. This description of the algorithm leads naturally to two other variants. We empirically evaluate these variants and also the implementation of the parallel algorithm.

The parallel architecture of general-purpose graphical processing units (GPUs) have been exploited for many real-world application in addition to applications in gaming and visualization problems.

GPUs have also been used to speed up RNA folding algorithms [5-7]. Here we show how the Four-Russians method allows an organization of the data structures for fast memory accesses. We also describe the organization of the parallel hierarchy to exploit the inherent parallelism of the solution.

In the rest of the section, we describe the problem in relation to the other problems in RNA folding. To keep the paper self-contained, we will first describe the *two-vector algorithm*, our application of the Four-Russians method to the RNA folding problem. We will use that description to describe the original Four-Russians method for RNA folding by Frid and Gusfield [4]. This discussion leads to two other variants where the preprocessing is done on demand, instead of the exhaustive preprocessing in the two-vector method and the Frid-Gusfield algorithm. In Section ‘An $O(\frac{n^{2}}{\log n})$ parallel algorithm we discuss the *O*(*n*^2^/ log*n*) parallel algorithm. We will then describe the implementation of a parallel algorithm using CUDA. The final sections have discussion on empirical observations and conclusions.

### Related work

The *O*(*n*^3^) dynamic programming algorithm due to Nussinov et al. [8,9] maximizes the number of non-crossing matching complimentary bases. There have been many methods since Zuker and Stiegler [10] that infer the folding using thermodynamic parameters [11,12] which are more realistic than maximizing the number of base pairs. These methods have been implemented in many packages including UNAFold [13], Mfold [14], Vienna RNA Package [15], RNAstructure [16].

Probabilistic methods include stochastic context-free grammars [17,18], the maximum expected accuracy (MEA) method, where secondary structures are composed of pairs that have a maximal sum of pairing probabilities, e.g., MaxExpect [19], Pfold [20], CONTRAfold [21] which maximize the posterior probabilities of base pairs; and Sfold [22], CentroidFold [23] that maximize the centroid estimator. There are also other methods that use a combination of thermodynamic and statistical parameters [24] and methods that use training sets of known folds to determine their parameters, e.g., CONTRAfold [21], Simfold [25] and ContextFold [26].

In addition to the Four-Russians method, other methods to improve the running time include Valiant’s max-plus matrix multiplication by Akutsu [1] and Zakov et al. [2]; and sparsification, where the branch points are pruned to get an improved time bound [27,28].

CUDA, the programming platform for GPGPUs, has been used to solve many bioinformatics problems. Chang, Kimmer and Ouyang [5] and Stojanovski, Gjorgjevikj and Madjarov [7] show an implementation of the Nussinov algorithm on CUDA. Rizk et al. [6] describe the implementation for Zuker and Stiegler method involving energy parameters. These methods are discussed later in the parallel implementation section.

## The Nussinov algorithm

In this paper, we consider the basic RNA folding problem of maximizing the number of non-crossing complimentary base pair matchings. Complimentary bases can be paired, i.e., A with U and C with G. A set of disjoint pairs is a matching. The pairs in a matching must not cross, i.e., if bases in positions *i* and *j* are paired and if bases *k* and *l* are paired, then either they are nested, i.e., *i* < *k* < *l* < *j* or they are non-intersecting, i.e., *i* < *j* < *k* < *l*. The objective is to maximize the number of pairings under these constraints.

The following algorithm, due to Nussinov [8] maximizes the number of non-crossing matchings. For an input sequence *S* of length *n* over the alphabet A, C, G, U, the recurrence is defined as follows. Let *D*(*i*,*j*) denote the optimal cost of folding for the subsequence from *i* to *j*. For all *i*, *D*(*i*,*i* - 1) = *D*(*i*,*i*) = 0 and for all *i* < *j*:

(1) $$D(i,j)=\max\left\{\begin{array}{l} b(S(i),S(\;j))+D(i+1,j-1)\\ \underset{i+1\leq k\leq j}{\max}D(i,k-1)+D(k,j) \end{array}\right)$$

where *b*(.,.) = 1 for complimentary bases and 0 otherwise. The DP table is the upper triangular part of the *n* × *n* matrix. The optimal solution is given by *D*(1,*n*). The table can be filled column-wise from the first column till the *n*^th^. There are other ways of filling the table too, e.g., along the diagonals — the (*i*,*i*)-diagonal first, (*i*,*i* + 1)-diagonal next and so on, until the last diagonal with one entry, *D*(1,*n*). To allow for traceback we need to store the bases that are paired to get the maximum value. Let *D*^∗^(*i*,*j*) denote the corresponding indices. These are obtained by substituting arg max in place of max in the above recurrence and can be computed along with the max value.

The first part of the recurrence can be solved in constant time. The second part is more expensive, incurring *Θ*(*n*) look ups and maximum computations. There are *O*(*n*^2^) entries in the DP table and each cell can be computed in *O*(*n*) time, giving an *O*(*n*^3^) time algorithm.

## The Four-Russians algorithms

In this section we discuss three variants of the Four-Russians algorithm. We will first describe the *two-vector* approach. Since it is simpler than the other methods we will use the description to discuss two other variants.

### Two-vector algorithm

To apply the Four-Russians technique we start with the following observation:

#### 

**Lemma 3.1. ***The values along a column from bottom to top and along a row from left to right are monotonically non-decreasing. Consecutive cells differ at most by 1*.

#### 

**Proof.** Consider the values of neighboring cells (*i*,*j*) and (*i* + 1,*j*). *D*(*i*,*j*) represents the solution of a longer sequence than *D*(*i* + 1,*j*). Therefore the former value should be at least as large as the latter. Suppose *D*(*i*,*j*) differed from *D*(*i* + 1,*j*) by more than one. Then we can remove any matching for *i*. This has at most one fewer base pair matching and is a valid solution for the subsequence (*i* + 1,*j*) with a larger value than its current value, contradicting the optimality of *D*(*i* + 1,*j*). An analogous argument holds along the rows. □

Once the cells *D*(*i*,*l*), *D*(*i*,*l* + 1), …, *D*(*i*,*l* + *q* - 1) are computed, for some *l* ∈ {*i*,…,*j* - *q*}, they can be represented by *D*(*i*,*l*) + *V*_0_, *D*(*i*,*l*) + *V*_1_, …, *D*(*i*,*l*) + *V*_*q* - 1_, where *V*_*p*_ = *D*(*i*,*l* + *p*) - *D*(*i*,*l*), for *p* ∈ {0,…,*q* - 1}. Let us define, *v*_0_ = 0 and *v*_*p*_ = *V*_*p*_-*V*_*p*-1_, for *p* ∈ {1,…,*q*-1}. From lemma 3.1, *v*_*p*_ ∈ {0,1}, for all *p* ∈  [ 0,*q*-1]. Let **v** denote the binary vector *v*_0_,*v*_1_,…,*v*_*q*-1_ of differences and let **V** denote the vector of running totals *V*_0_,*V*_1_,…,*V*_*q*-1_.

Since the *v*_*p*_’s are defined from *V*_*p*_’s, the inverse function is well defined: $V_{p}=\sum_{k=0}^{p}v_{k}.$ Thus *D*(*i*,*l*) together with the vector **v** represents *q* consecutive cells of the table.

Similarly, since the values are non-increasing down a column, *D*(*i* + *l* + 1,*j*),…,*D*(*i* + *l* + *q*,*j*) be represented by the pair $D(i\;+\;l\;+\;1,j),\bar{v}$, where $\bar{v}\in {\left\{0,-1\right\}}^{q}$. The corresponding vector of cumulative sums is denoted $\bar{V}$. We call **v** the *horizontal difference vector* or the *horizontal vector* and we call $\bar{v}$ the *vertical difference vector* or the *column vector*.

Consider *q* consecutive cells from *l* + 1 to *l* + *q* used in computing *D*(*i*,*j*):

(2) $$\begin{array}{lcl} D(i,j) & \leftarrow & \underset{l+1\leq k\leq l+q}{\max}\;D(i,k-1)+D(k,j)\\ & \leftarrow & \underset{0\leq k\leq q-1}{\max}\;D(i,l)+V_{k}+D(i+l+1,j)+{\bar{V}}_{k} \end{array}$$

(3) $$\begin{array}{lcrrr} & & & \;\;\;\;\leftarrow & \;D(i,l)+D(i+l+1,j)+\underset{0\leq k\leq q-1}{\max}\;\;\;V_{k}+{\bar{V}}_{k} \end{array}$$

As before, we use arg max in place of max to obtain *D*^∗^(*i*,*j*), which facilitates the traceback.

As noted above the second line of the recurrence (1), looping over elements, is more expensive part of the computation and we will use (3) instead of (2) to compute the *D* and *D*^∗^ values in the Four-Russians method. That is, we will use (3) over groups of *q* cells each instead of one loop of (1). Since the *V* vectors are in bijection with the **v** vectors, we will use **v** in the computation. Let **v** and $\bar{v}$ be the corresponding vectors in (3). The following algorithm evaluates the max computation.

Using this instead of (2) is not advantageous in itself. However, if this algorithm is given as a black box, *D*(*i*,*j*) can be computed in constant time by invoking the black box once. To exploit this fact, we will preprocess this computation over all possible **v** and $\bar{v}$ vectors and tabulate the results in *R*. Table *R* is indexed by a pair of numbers in the range [2^*q*^] to represent the two vectors $\left(v,\bar{v}\right)$. The table lookup is a constant time operation as it fetches the max and arg max values. We will show later that this exhaustive enumeration is not too expensive.

In the Nussinov algorithm described in the previous section, the recurrence over *q* cells is evaluated using (2) and it takes *O*(*q*) time. In the Four-Russians method, using the preprocessing step, the max computation is available through a table lookup and the recurrence for *q* terms can be completed in constant time. This reduction in the computation time is the reason for the speedup by a factor of *q*.

The two-vector method modifies the Nussinov algorithm as follows. All the rows and columns of the table are grouped into groups of *q* cells each. The recurrence over these *q* cells is computed in constant time using the preprocessing table. The recurrence involves *D*(*i*,*k* - 1) + *D*(*k*,*j*), i.e., the value in the (*k* - 1)^st^ column is used with the *k*^th^ row. Therefore the row and column groupings differ by one. That is, the columns are grouped (0,1,…,*q* - 1), (*q*,*q* + 1,…,2*q* - 1) etc. The rows are grouped (1,2,…,*q*), (*q* + 1,*q* + 2,…,2*q*) etc. This ensures that the row and column groups are well characterized. That is, to fill the cell (*i*,*j*), the *k*^th^ group along row *i* needs to be combined with the *k*^th^ group below (*i*,*j*) in column *j*.

**Figure 1:**
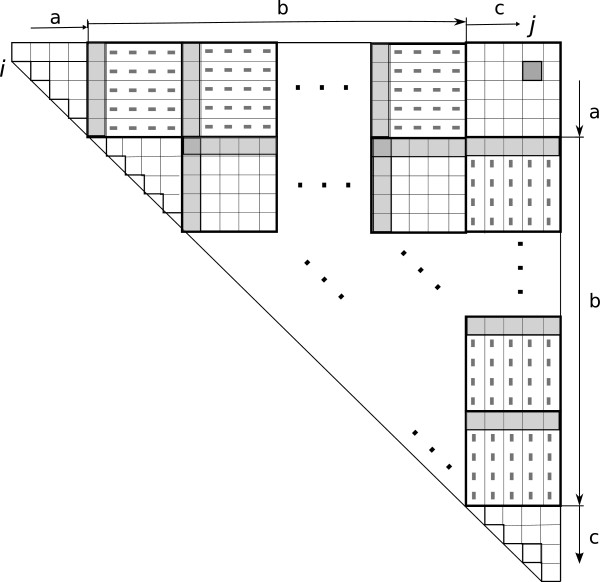
**A diagrammatic representation of the two-vector method.** The row and column blocks are matched as labelled. The gray boxes and the gray dashes show the initial value and difference vectors. The group of cells in *b* correspond to the Four-Russians loop in lines 16–20 of Algorithm 1; the cells in *a* are used in the loop in lines 10–12 and the cells in *c* form the loop in lines 13–15.

The cells of the table are filled in the same order as before. When the last cell of a row- or a column- group is evaluated the corresponding row and column vectors are computed and stored. To fill cell (*i*,*j*), we retrieve the first element and the horizontal vector of the group from row *i* and the first element and the column vector from the corresponding group in column *j*. The recurrence is solved using (3) by a table lookup. The final value for *D*(*i*,*j*) is the maximum value over all the groups. There might be residual elements in the row that do not fall in these groups. There are at most 2*q* such elements. These are solved separately using Nussinov’s method. Algorithm 1 has the algorithm listing and Figure 1 describes the algorithm pictorially.

**Algorithm 1** Procedure for the two-vector Four-Russians speedup. The DP table is filled column-wise.

#### Runtime analysis

In the precomputation phase, there are 2^*q*^*q*-length vectors and 2^2*q*^ pairs of vectors. The precomputation takes *O*(*q*) time per vector pair. Thus the total time for precomputation is *O*(*q*2^2*q*^).

The main algorithm: There are *O*(*n*^2^) cells and to fill each cell it takes *O*(*n*/*q* + *q*) time. That is, it takes *O*(*n*/*q*) time to look up the initial value and the difference vector and the *R* table lookups for the the *O*(*n*/*q*) groups. It takes *O*(*q*) time for the residual elements. Thus it takes *O*(*n*^2^ × (*n*/*q* + *q*)) time to fill the table. Every cell is involved in at most two vector computations, where the difference to its neighbor is computed once for the row and for the column vector. This takes an amortized *O*(*n*^2^) time which is dominated by the rest of the algorithm.

When *q* = log*n*, the total time for the entire algorithm is $O(\log n\;2^{2\log n}\;+\;n^{2}\;+\;n^{2}\times (\frac{n}{\log n}\;+\;\log n))=O(n^{2}\log n\;+n^{3}/\log n)=O(n^{3}/\log n)$.

### FG algorithm

Frid and Gusfield [4] first showed how the Four-Russians approach could be applied to the RNA-folding problem. We will call their algorithm the *FG* algorithm. *FG* and *two-vector* algorithms are variants of the same idea. We will highlight the differences in preprocessing and the maximum value computation by the Four-Russians technique. In particular, we will show the maximum computation in step 19 of Algorithm 1.

After computing the *q*-contiguous cells of a group in a row, the value in the initial cell *D*(*i*,*p*) and the horizontal difference vector *v*_*p*_ are known. They run the preprocessing algorithm in page 3 for this fixed *v*_*p*_ vector together with all possible vertical difference vectors. They add the value of *D*(*i*,*p*) to the maximum and table the result. This preprocessing step is computed for every block of every row. The preprocessing table *R* is indexed by row number, group number and a vector (which is a potential column vector). The horizontal vectors need not be stored.

Notice the difference between the two-vector and the FG variants. In two-vector algorithm the preprocessing for vector pairs is computed once for each distinct pair of vectors. Whereas, in FG the preprocessing step is run once for each group of each row, even if the vector pair was seen earlier. This is because the table contains the result of addition of the initial cell of the group *D*(*i*,*p*).

To fill cell (*i*,*j*), they iterate over all groups and find the *q*-length column vectors. The preprocessed value for this vector in the corresponding block is retrieved from the table and the result is added to *D*(*q*,*j*).

The preprocessing is for horizontal vectors seen in the table. Since the horizontal vectors are not known beforehand, the precomputation cannot be done prior to the main algorithm. Instead, it is interleaved with the computation of the table. They fill part of the DP table and use the vectors to complete some preprocessing, which in turn is used fill another part of the table and so on.

Since the preprocessing is done for every group of every row, the same horizontal vector can be seen multiple times in the table. This leads to duplicated work and slower running time than the two-vector algorithm.

The running time for the FG method is $O(\frac{n^{3}}{\underset{b}{\log}\;n})$, where 2 < *b* < *n* and *b*^*q*^=*n*.

### Two variants that memoize

The two-vector method computes the preprocessing over all possible vector pairs. While the computation is for done exactly once for the unique vector pairs, some of these vector pairs might not be seen in the table. In the FG method, the precomputation step is for only the horizontal vectors that are seen in the table. However, for some vectors, the computation is duplicated. Stated this way, a hybrid approach suggests itself.

In our next variants, we memoize the results for a pair of vectors. Like the two-vector approach, the preprocessing is done only once for a vector pair and like the FG algorithm, it is only for the vectors seen in the table and the preprocessing is interleaved with the main algorithm. Since the preprocessing table is indexed by two vectors, unlike the FG algorithm, the results are computed only once for every vector seen.

In the partially memoized version, upon completion of elements of a group, if a new horizontal vector is seen, we pair it with all possible 2^*q*^ column vectors and the results are tabled. In the completely memoized version, the result for a pair of horizontal and vertical vectors are computed the first time the pair is observed and the result is stored in the table. The computed values for retrieved from the table when they are seen again. The rest of the algorithm is identical to the two-vector method.

All these variants take *O*(*n*^3^/ log*n*) time but the memoized versions potentially store fewer vectors than the two vector method and will have a similar worst-case runtime in practice as the two-vector method. But, as argued before, the FG method does duplicated work and will be slower in practice.

## An $O(\frac{n^{2}}{\log n})$ parallel algorithm

The Nussinov DP algorithm can be parallelized with *n* processes to get an *O*(*n*^2^) parallel algorithm on a concurrent-read concurrent-write parallel random access memory (CRCW PRAM) machine. In the parallel algorithm, we fill the table diagonal by diagonal. We use *n* processes and assign one parallel process to each column. In the *i*^th^ iteration, the *p*^th^ process computes the value for the (*p*-*i*)^th^ diagonal entry. That is, in the first iteration, the bottom-most cell in each column, i.e., the entries in the main diagonal are solved and in successive iterations, the diagonals above are solved. To compute the value for cell (*i*,*j*), the entries in the row to its left and in the column below (*i*,*j*) are needed. The entries in the same column are computed in earlier iterations. Similarly, the entries on the left are solved by other processes in earlier iterations. Since these values are computed in earlier iterations, each diagonal cell can be filled independent of the other processes.

More formally, we have *j* ∈  [ *n*] parallel processes and the *j*^th^ process computes the values of values along the column *j*. All processes synchronize after filling an entry; this ensures that values needed to fill a cell are computed by the other processes.

The parallel algorithm for process *j* for *j*=1,2,…,*n*:

A process has to compute the value for *O*(*n*) cells and for each cell it needs to access *O*(*n*) other cells. Thus the total computation takes *O*(*n*^2^) time with *n* processes.

We will describe the use the two-vector Four-Russians method to obtain an *O*(*n*^2^/ log*n*) algorithm below. The preprocessing step that enumerates the solution for 2^*q*^ × 2^*q*^ difference vectors is embarrassingly parallel and we do not discuss the parallel algorithm for it.

As before, we fill the table along the diagonals. We use *n* processes, one for each column. Each process solves the entries of the column from bottom to top. As in the serial algorithm, computing the maximum by looping over all the entries is the expensive part of the computation and will be optimized. Instead of looping over the individual entries (lines 4 – 6 in the parallel algorithm above), we use the Four-Russians technique to solve *q* cells in one step by looking up the table computed in the preprocessing step.

Let *d*_*H*_(*i*,*j*) be the horizontal difference vector for cells *D*(*i*,*j*),…,*D*(*i* + *q* - 1,*j*) and let *d*_*V*_(*i*,*j*) be the vertical difference for cells *D*(*i*,*j*),…,*D*(*i* + *q* - 1,*j*). We modify the inner loop of the parallel algorithm as follows:

For each entry, the first loop takes *O*(*n*/*q*) time and the second loop takes *O*(*q*) time. Since all the processes are solving the *k*^th^ diagonal in the *k*^th^ iteration, all of them execute the same number of steps before synchronization. Note that we compute the horizontal and vertical differences for every node, unlike in Algorithm 1 where they are computed every *q*^th^ cell, to ensure that every process performs the same number of steps and simplify the analysis. The difference vectors can be computed in *O*(*q*) time. These can also be computed in constant time by shifting the previous difference vector and appending the new difference. But we will not assume this simplification for the time bound computation.

Thus each entry can be computed in *O*(*n*/*q* + *q*) time. There are *O*(*n*) entries for each process, thus the total time taken for all processes to terminate is *O*(*n*^2^/*q* + *nq*). With *q* = log*n* as before, this gives an *O*(*n*^2^/ log*n*) algorithm.

## Parallel implementation

### GPU architecture

Graphics processing units (GPUs) are specialized processors designed for computationally intensive real-time graphics rendering. In addition to manipulating graphics objects the parallel architecture can be exploited for other tasks where large amounts of data are to be processed in parallel. Compute Unified Device Architecture (CUDA) is the computing engine designed by NVIDIA for their GPUs. It allows the programmer to write highly parallel code and provides platform-specific optimizations.

In CUDA, a serial program on a “host” CPU launches parallel “kernels” on the “device” GPUs. Kernels specify the code to be executed by all the threads. Every thread executes the same code in a kernel but can be assigned a different part of the task based on their indices. This paradigm is called Single-Instruction Multiple-Thread (SIMT), which is similar to Single-Program Multiple Data (SPMD) where the threads are run almost in lockstep.

The programmer can group threads in a *block*, which in turn can be organized in a *grid* hierarchy. The threads in a block and blocks in a grid can be organized in one-, two- or three-dimensions. While the hierarchy is specified when launching a kernel, thread management is handled by the underlying system. The threads within a block use barrier synchronization. Different blocks communicate by atomic memory operations in global memory.

Memory hierarchy includes thread-specific local memory, block-level shared memory for all threads in the block and global memory for the entire grid. The access times increases along the hierarchy from local to global memory.

Kernels are very fast when threads run in lockstep. If certain threads take a conditional branch or are delayed by memory access then all the threads in the block are stalled. Since the access to global memory is slow (more clock cycles than local memory access), it is efficient for the threads within a block to access contiguous memory locations. Then the hardware *coalesces* memory accesses for all threads in a block into one request. More specifically, in our application, if a matrix is stored in row-major order and if the threads in a block access contiguous elements of a row, then the accesses can be coalesced. Whereas accessing elements along a column is inefficient as distant memory elements have to be fetched from different cache lines.

Programs that observe the hardware specifications can exploit the optimizations in the system and are fast in practice. We designed the program that exploits the parallel structure of the DP algorithm and the hardware features of the GPU.

### Related work

As mentioned earlier, the cells of a diagonal are independent of one another and can be computed in parallel. In Stojanovski et al. [7], elements of the diagonal are assigned to a block of threads. This design does not handle memory coalescence for either row or column accesses. Chang et al. [5] allocate an *n* × *n* table and reflect the upper-triangular part of the matrix on the main diagonal. Successive elements of a column are fetched from the row in the reflected part of the matrix. When threads of a block are assigned to elements of a diagonal, the successive column accesses for a thread are to consecutive memory cells. However, this does not allow coalesced access for threads within a block. Rizk and Lavenier [6] show an implementation for RNA folding under energy models. They show a tiling scheme where a group of cells are assigned to a block of threads to reuse the data values that are fetched from a column. In this paper, we show that storing the row and column vectors in different orders for two-vector method can further improve the efficiency.

### Design of the Four-Russians CUDA program

#### *The high-level idea*

Like in the parallel algorithm described in Section ‘An $O(\frac{n^{2}}{\log n})$ parallel algorithm’, we will fill the cells of the table along the diagonals. However, for efficiency reasons, we cannot fill each diagonal in order. For example, by assigning different cells of a diagonal to a block of threads, each thread has to access a different row and different column. This design is not efficient as threads in a block try to access different locations of global memory which slows down the program. For the program to be efficient on the CUDA architecture, we exploit coalesced memory access for faster computation and ensure that individual threads are not stalled. We create a tiled table by grouping *q* × *q* cells into a tile and assign a block of threads to fill a tile. We will show that the tiles are independent and assign a grid of blocks to solve a diagonal of tiles in parallel. Thus the implementation can be thought of as a generalization of the Four-Russians algorithm on the tiled table. The details follow.

#### *Data structures*

Notice that cells *D*(*i*,*j*) and *D*(*i*-1,*j*) both use values in column *j* below *D*(*i*,*j*). Similarly, *D*(*i*,*j* + 1) and *D*(*i*,*j*) refer to values in row *i* to the left of cell (*i*,*j*). By grouping these cells together and assigning this group to a block of threads the values along the rows and columns should be fetched once per block. We will group *q* × *q* cells together and store the values from the rows and columns in shared memory. As noted earlier, for the four-Russians method, it is convenient to group the rows *kq* + 1,…,(*k* + 1)*q* and columns *kq*,…,(*k* + 1)*q*-1, for *k* ∈ {0,…,⌊*n*/*q*⌋}. We now have a *tiled table*, where each tile is a composite of *q* × *q* cells. The tiles along a diagonal can be computed independent of each other. Each tile is assigned to a block of threads and computed in parallel. After all the entries of the tile are computed, only the horizontal and vertical differences are stored. The horizontal and vertical difference vectors are used by the Four-Russians technique for later computations.

To fill a tile, the horizontal differences of all the tiles to the left and vertical differences from the tiles underneath are accessed. By storing these difference vectors together the memory accesses can be coalesced. That is, we store the horizontal differences of the rows in a tile together. Similarly, the vertical differences of the tile are grouped together. However, the horizontal and vertical differences of the tiles are stored in different order. The horizontal differences are stored in row-major order and the vertical differences are stored in column-major order to exploit coalesced memory access.

When each thread retrieves one vector from a tile, the block of threads accesses contiguous memory locations and the memory accesses are coalesced. Successive iterations fetch the vectors from tiles along a row which are in contiguous memory locations. Similarly the vertical differences of a tile below are accessed in one coalesced memory access by the threads of the block.

Since we group *q* elements together, at the last column of tiles might have fewer than *q* elements and handling the corner cases at the GPU will involve extra checks which might slow the program down. We can avoid these by padding the sequence with extra characters. The modified string has *n*^′^=*n* + *q*-*n* mod *q* characters. The final result is still stored in cell *D*(1,*n*).

#### *Thread hierarchy*

There are a few options for assigning threads to compute various cells in a tile. We can assign *q*^2^ threads, one per cell, or assign *q* threads, one per row or column. While it appears that *q*^2^ threads admit more parallelism, using *q* threads is more advantageous for a number of reasons.

There is a limit on the number of threads that can be allocated on a CUDA device. Requesting *q*^2^ threads per kernel might limit the number of kernels that can be launched in parallel. At various synchronization points and in conditional branches (like updating a new maximum value in a cell) all threads are stalled. Moreover, with *q*^2^ threads, most of the threads will remain idle for many iterations due to the dependency on other cells. Instead, the *q* threads can be organized to keep the threads active in most iterations and perform the same computations with fewer stalls.

### The algorithm

Let *N* be the number of diagonals in the tiled table (*N* = (*n*^′^ + 1)/*q*). The main algorithm run on the CPU is as follows. The various kernels are described next.

We will first describe simple_kernel. The other kernels have a similar structure and are described next. The *j*^th^ thread solves the *j* column for all rows of the tile. It fetches the horizontal difference and initial cell value of the *j*^th^ row of a tile to the left and stores it in shared memory. The other threads in the block fetch from the other rows of the tile to shared memory. These reads occur in parallel and are coalesced as they refer to contiguous memory addresses. The *j*^th^ thread then fetches the vertical difference and initial value of the *j*^th^ column of the corresponding tile below. Since only the *j*^th^ thread needs this data, it stores it in local memory.

The threads use these values and compute the values for the all the rows. This is repeated for all tiles. This corresponds to the loop in lines 16–20 of Algorithm 1. Finally to complete filling the tile, the rows are filled from bottom to top. At every row, line 5 and the loop in lines 10–12 are executed in parallel. Then the loop in lines 13–15 are executed. The threads cannot independently to complete this loop. The threads use synchronization to ensure that the values in the columns to the left are written before they are used. Finally, the *j*^th^ thread computes the horizontal differences of the *j* row and the vertical differences of the *j*^th^ column and store the values in the corresponding memory locations.

#### *The parallel kernels*

For every diagonal element, the Four-Russians part of the code has to fetch the values from tiles below and to the left. Closer to the top of the matrix, there are more tiles to retrieve data from. Since these tiles are all independent, the respective computations can be parallelized. For the *k*^th^ diagonal, we launch ⌊*k*/*p*⌋ kernels per tile. Each kernel will iterate over *p* tiles and store the result in a temporary location. We will then launch another kernel which will iterate over the remaining tiles and then complete the rest of the steps like simple_kernel to compute the values of the individual cells of the tile and store the horizontal and vertical differences.

#### *Initial diagonals*

The *i*^th^ block of the init_diag kernel will solve the (*i*,*i*)^th^ diagonal tile and (*i*,*i* + 1)^th^ tile. The values in the (*i* + 1,*i* + 1)^th^ diagonal tile are also needed to solve the (*i*,*i* + 1) the tile. Instead of waiting for a different kernel to solve it and then read it from global memory, those values are also solved by the *i*^th^ block locally but the results are not stored. The (*i* + 1)^th^ block also computes these values and stores the horizontal and vertical differences.

The values for these tiles are computed by standard Nussinov algorithm. To solve the (*i*,*i* + 1)^th^ tile, the *j*^th^ thread solves the *j*^th^ diagonal like in simple_kernel.

## Empirical results

Prior to empirical evaluation, the FG algorithm was expected to be the slowest due to the repeated computation. The memoized versions were expected to be faster than the two-vector algorithm, as they preprocess only a subset of the 2^2*q*^ vectors seen in the table.

We ran the programs on complete mouse non-coding RNA sequences. We also tested the performance on random substrings on real RNA sequences and random strings over A,C,G,U.

The FG algorithm, while faster than Nussinov, was the slowest among the Four-Russians methods, as expected. The completely memoized version was slower than the other two variants. This is because every lookup of the preprocessing table includes a check to see if the pair of vectors has already been processed. There are 2^2*q*^ unique vector pairs but there are $O(\frac{n^{3}}{q})$ queries to the preprocessing table and each query involves checking if the vector pair has been processed plus the processing time for new pairs. There are $O(\frac{n^{2}}{q^{2}})$ vector pairs in the table. For larger *n* (e.g., *n*>1000 and *q*=8), all the 2^2*q*^ vectors are expected to be present in the DP table. Generally, memoized subproblems are relatively expensive compared to the lookup. Since the preprocessing here has only *q* steps, the advantage of memoization is not seen.

The partially memoized version was slightly slower than the two vector algorithm. Again, the advantage of potentially less preprocessing than the two-vector method is erased by the need to check if a vector has been processed. The two-vector method was the fastest on all sequence lengths tested.

**Figure 2:**
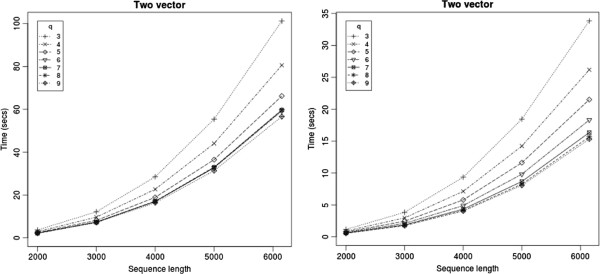
Running time of the two-vector method on a desktop and on a fast server.

For short sequences the two vector method took negligible time (less than 0.2 seconds up to 1000 bases) and are not reported. For longer sequences, we noticed that using longer vector lengths reduced the running time. However the improvement saturated at *q* = 8 or 9 (Figure 2). Beyond this, the extra work in preprocessing overshadowed the benefit. A similar trend was seen for the memoized versions too. However, for the FG method *q*=3 gave the best speedup and longer vector lengths had a slower running time due to the extra preprocessing at every group.

The algorithms implemented compute the same matchings as the Nussinov algorithm. The correctness of each implementation was evaluated by comparing the entried of the DP table of the Nussinov algorithm to the analogous values computed by the faster implementation. All the programs were written in C++ compiled with the highest compiler optimizations. We only discuss the experimental results on a desktop and two GPU cards in this paper.

We measured the running times of the different versions of our serial algorithms on a desktop machine with a Pentium II 3 GHz processor and 1 MB cache. The running times of Nussinov and the speedups of various programs compared to Nussinov are shown in Table 1.

**Table 1:** Speedup factors of the serial programs on the desktop

	**Time**	**Speedup**			
**Length**	**Nussinov**	**Two-vector**	**Partially**	**Completely**	**FG**
	**(in secs)**		**memoized**	**memoized**	
2000	16.5	7.7	7.3	5.6	3.0
3000	62.5	8.8	8.3	6.4	3.4
4000	196.6	11.9	11.4	8.8	4.7
5000	630.3	21.1	18.9	14.7	7.8
6150	1027.8	18.1	17.0	13.3	7.03

The times reported are an average over 10 sequences of approximately the same lengths. Among the serial programs tested, FG had the slowest running times and two-vector method had the best running times. For sequences of length 6000, the two-vector method takes close to a minute on the desktop. As discussed earlier, the extra steps to check if a vector or a pair of vectors have already been processed takes longer than the benefit of potentially fewer steps needed for preprocessing.

On sequences of length 5000 bases, two vector had a 20 times speedup, and FG has a 7 times speedup over the Nussinov program on a desktop machine with a Pentium II 3 GhZ processor and 1 MB cache. On a server with four 64-bit cores of Xeon 2.8 GhZ machines with 8 MB primary cache, the running times of all the programs were faster, but the relative speedup on Nussinov was more drastic than that of other programs. The same relative trends for the Four-Russians programs were seen — two-vector method was faster than the partially memoized problem; the totally memoized version, while faster than the FG algorithm was slower than the other two variants. Figure 2 shows the running times of the two-vector method on the desktop and servers, respectively.

**Figure 3:**
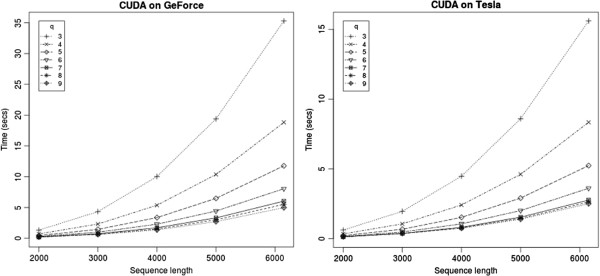
**Running time of the CUDA program on two GPUs.** The programs run twice as fast on the Tesla card than the GeForce card.

Figure 3 shows the execution times on two GPU cards – GeForce GTX 550 Ti card with 1 GB on-card memory and Tesla C2070 with 5 GB memory. The programs take about a second for sequences up to 4000 bases long, and takes about 5 seconds and 2.5 seconds for sequences of length 6000. The running times for various sequence lengths are shown in Table 2. Even in the parallel implementation, we see the same trend with increasing the vector lengths — there is a marked decrease in running time by increasing the vector lengths from *q*=3, and the improvement saturates around *q*=8 (Figure 3).

**Table 2:** Running times for the parallel program (in secs)

**Length**	**On GeForce**	**On Tesla**
2000	0.20	0.14
3000	0.62	0.38
4000	1.36	0.74
5000	2.70	1.39
6000	4.97	2.50

Details on running times of the other variants can be found in the technical report [29].

## Conclusions

We described the two-vector method for using the Four-Russians technique for RNA folding. This method is simpler than the Frid-Gusfield method. It also improves the bound of the parallel algorithm by a log*n* factor to $O(\frac{n^{2}}{\log n})$. We showed two other variants that memoize the preprocessing results. These methods are faster than Nussinov by up to a factor of 20 and the Frid-Gusfield method by a factor of 3.

In the future, it will be interesting to see the application of the Four-Russians technique for other methods that use energy models with thermodynamic parameters. The Frid-Gusfield method has been applied to RNA co-folding [30] and folding with pseudoknots [31] problems; the application of the two-vector method to those problems and its implications are also of interest. It will be interesting to compare our run time with the other improvements over Nussinov, like the boolean matrix multiplication method [1].

## Competing interests

The authors declare that they have no competing interests.

## Authors’ contributions

BV designed the two-vector algorithm, the memoized algorithms, the parallel algorithm. He also designed and implemented the programs and drafted the manuscript. DG motivated the problem and helped in improving the manuscript. DG and YF designed the FG algorithm. All authors read and approved the final manuscript.
